# Assessment of Food Quality in School Canteens: A Comparative Quantitative Study between Primary and Secondary Schools in Malaysia

**DOI:** 10.3390/nu13093009

**Published:** 2021-08-28

**Authors:** Leng Huat Foo, Ying Jing Tan

**Affiliations:** School of Health Sciences, Health Campus, Universiti Sains Malaysia, Kubang Kerian 16150, Kelantan, Malaysia; yingt3200@gmail.com

**Keywords:** school food environment, food availability, food quality, schools

## Abstract

Schools are an important food environment to cultivate and promote healthy food choices and practices among children and adolescents. The aim of the present study was to assess the type and quality of food and beverages sold in school canteens in public primary and secondary schools in Kelantan, Malaysia. Eligible schools were randomly selected from the list of all schools and detailed information of all food and beverage items sold in the school canteens were collected during school days. Food and beverages were classified based on food groups derived from the Malaysian Food Dietary Guideline and the Recommended Foods for Healthy Cafeteria Guideline. An assessment of the traffic-light nutrition food-labelling system of the total sugar content in all pre-packaged foods was also undertaken. A total of 568 food items were identified, with secondary school canteens selling a greater proportion of food items than the primary schools (55.5% vs. 44.5%). In terms of the main food groups, grains and cereal products represented the largest food group served (33–36%), followed by beverages (21–25%) and confectionary and sweet foods (12–13%). In contrast, the vegetable and fruit group represented the smallest proportion of food items sold (1–3%). Comparisons between primary and secondary schools showed a similar trend and pattern of food types and quality of foods sold, except for animal-based foods. A greater percentage of food items in this category was found among secondary schools (12.1%) versus primary schools (6.7%). When total sugar content of all pre-packaged foods was quantified based on the traffic-light nutrition-labelling system, almost one-third of foods and beverages were classified as high (29.1%). Confectionary (19.1%) and flavoured milk and fruit drinks (10.0%) both exceeded the recommended sugar levels of >22.5 g per 100 g and >11.25 mL per 100 m L, respectively. Only one of these packaged foods and beverages (0.9%) was classified as a healthy food choice. About a quarter of the food items available in school canteens were classified as prohibited based on a new revised list of prohibited food and beverage items. These findings indicate that, despite the Guidelines, a large number of unhealthy food items are being sold in school canteens. Hence, interventions such as sustainable healthy school canteen menus should be implemented to promote healthy food choices amongst school-aged children.

## 1. Introduction

Childhood obesity has emerged as one of the major global public health crises, including in Malaysia where the prevalence of childhood obesity has markedly increased over the years [1]. Childhood obesity causes a wide range of psychosocial and health consequences during the growing years and, if untreated, also across adulthood [1,2,3,4]. Based on the recent Malaysian nationwide survey in 2019, the prevalence of overweight and obesity increased from 15.1% in 2011 to 29.8% in 2019 among children aged 5–17 years [5]. This finding highlights the dramatic increase in the prevalence of childhood obesity in Malaysia with attendant impact on psychosocial and cardio-metabolic health later in life.

It is well-documented that a poor diet is one of the major determinants of an increased risk of malnutrition from underweight to obesity in school-aged children [6,7]. Considerable research to date has been undertaken to assess and identify the underlying causes and determinants of food choices and eating behaviours [8,9,10,11], in which multiple factors ranging from individual biological, genetic, psychosocial and lifestyle factors, family and social influences, institutional, neighbourhood and community environments, and macro-system influences, such as marketing, culture, and values within the food systems, may directly or indirectly influence a child’s eating behaviours [12].

A growing body of evidence suggests that other factors, such as the food environment and its availability and accessibility, play an important role and is often referred to as the ecological behaviour model [13,14,15]. For example, access and availability to healthy foods at home is significantly associated with eating behaviours and better diet quality [14,16]. In contrast, access to unhealthy food outlets around homes and/or within the wider community, such as fast food restaurants and convenience stores, are associated with an increased risk of excessive weight gain [13,15,17]. These findings have highlighted the importance of the relationship between food choice and the environment [18].

The school food environment has a potentially significant impact on a child’s dietary practices as they spend more time in school than in any other environment away from home and consume almost half of their total daily energy in the school setting [19,20]. Hence, school should be considered one of the key environments where healthy eating behaviours could be established, and at the same time, childhood obesity could be tackled [20,21,22]. Numerous studies from the United States [23] and Australia [24] have shown that higher availability of less-healthy food and beverages, high in energy, sugar, fats, and salt, in schools was associated with poor nutritional status and higher energy intake. Despite the growing body of evidence from Western countries, school food environments are quite different across Asia in general, and particularly in Malaysia, hence results cannot be directly extrapolated. To the best of our knowledge, very limited work has been conducted in Asia to investigate food types including cooked and pre-packaged foods and their quality in school canteens. Moreover, an assessment of the school food environment would help us to understand and identify the quality of food and beverages available and their association with poor health [19]. In turn, a better understanding of the food and beverage available within schools may have a broader impact on eating behaviours and future disease risk [20]. Therefore, the aim of the present study was to assess the availability, type, and quality of food and beverages available in both primary and secondary school canteens in Kelantan, Malaysia. We also assessed the total sugar content of the pre-packaged food and beverages using a traffic-light nutrition classification system and assessed the proportion of these food items sold in relation to the Healthy School Canteen Management Guide, Ministry of Education Malaysia [25].

## 2. Materials and Methods

### 2.1. Study Design

A list of all schools in the district was obtained from the State Department of Education, Ministry of Education, Malaysia. Only schools that met the inclusion criteria of being government-funded with non-religious and special educational needs, were eligible to be included in the study as the main national education system provided to most of the students in Malaysia. A total of 140 public schools, 97 and 43 primary and secondary schools, respectively, were eligible to be included in the study. These schools were further stratified into two main categories namely, national school (135 schools) and national-type Chinese school (vernacular school) (5 schools), as reflected by the ethnic composition of the population in Kelantan. Initially, a total of 12 public schools (6 schools for each of primary and secondary categories), were recruited with a simple random sampling ratio of national school to national-type Chinese school of 2:1 to represent the student population distribution by ethnic groups in Kelantan. However, all schools suspended operations due to the implementation of the nationwide Movement Control Order associated with the COVID-19 pandemic on 18 March 2020. Hence, a total of 10 schools, comprising three national schools and two national-type Chinese schools from both primary and secondary categories were included in the final analysis. Once all eligible schools were identified, a letter of invitation, including study information sheet such as the study purpose and procedures, was given to school principals. Informed consent was obtained from school authorities and food operators prior to data collection. Data collection, using direct observation of all foods sold in school canteens, was conducted between January 2020 and March 2020. As the present study did not include human participants; no research ethics approval was required.

### 2.2. Measurements

All information of foods and beverages in various preparation forms such as pre-packaged foods, ready-to-eat cooked and pre-ordered cooked meals in terms of the ingredients used and portion serving served for each meal were collected by researchers and helpers across a number of days to minimise the effect of intra-day food variation. Information was double-checked with school canteen menus provided. In addition, images of a whole plate or whole bowl or whole tray of cooked food were taken to capture the actual portion and size of the food and beverage served. For ordered cooked meals served in school canteens, images were taken after the food was cooked and served, during recess time. For pre-packaged foods, information regarding total calories, fat, sugar, and salt listed on the front-of-pack nutrition labels were also collected.

### 2.3. Classification of Measurement Variables

All food and beverage items sold in school canteens were classified into 10 main food groups (vegetables, fruits, grains and cereal products, eggs, nuts and legumes, animal-based foods, milk and dairy products, snack and fast foods, confectionary and sweet foods, and beverages), as most main food components were included based on the current revised Malaysian Dietary Guidelines [26].

We used the UK-based front-of-package Nutritional Traffic Light rating guidelines for total sugar content per 100g of food (or per 100 mL for beverages) (Table 1), developed by the Food Standards Agency (FSA), UK, in 2006 [27]. Total sugar content of all pre-packaged foods and beverages is classified as a low- (green traffic light, healthiest), medium- (amber traffic light) or high-sugar food (red traffic light, least healthy). In addition, the extent and degree to which these pre-packaged foods were further classified based on the NOVA food processing classification criteria into (i) unprocessed or minimally processed foods, (ii) processed culinary ingredients, (iii) processed foods, and (iv) ultra-processed food and drink products [28].

The quality of foods and beverages available were further compared with the two different approaches, namely, (i) the revised list of prohibited food and beverages items sold in school canteens by the Healthy School Canteen Management Guide [25] and (ii) the highly recommended healthy food choices menu offered by the Healthy Cafeteria Initiative Program [29]. In brief, numerous food and beverages that are high in fats, sugars, and salt contents such as instant noodles, cakes and donuts, fried crackers, and processed foods, such as burgers, sausages, nuggets, sweet chocolate snacks, candy, junk foods, cream roll bread, creamy biscuits, processed pickled foods, pre-mixed cordial and syrup drinks, and carbonated flavoured drinks, have been considered prohibited food and beverage items [25]. In addition, there are several proposed food types and choices such as grains and cereal-based foods, wholemeal or wholegrain breads, vegetables, fruits, seafood, poultry and eggs, legumes, and low fat milk or skimmed milk, that are highly recommended in the Healthy Cafeteria Initiative programme conducted by the Ministry of Health Malaysia [29].

### 2.4. Statistical Analysis

Descriptive statistics were generated for characteristics of all food and beverage items, as expressed in mean and standard deviation for continuous variables and percentages and numbers for categorical variables. In addition, food and beverages that appeared multiple times in the same school were coded only once to prevent the over-counting of similar food. There was no formal statistical test needed, as the number of potential comparisons was very large and there was no clear mechanism by which the multiple possible dimensions of testing for differences could be represented and/or tested.

## 3. Results

Table 2 shows the general characteristics of the types of food and beverages available in both primary and secondary schools in the study. A total of 568 food items were identified for both schools, with secondary school canteens selling a greater proportion of food items than the primary schools (55.5% vs. 45.5%). Comparisons of the food and beverage distributions between primary and secondary schools showed that there was a quite consistent trend and pattern in almost all food types, except for pre-packaged foods that were more common in the secondary school canteens than in primary school canteens (15.6% vs. 7.5%). Ready-to-eat cooked food was the most common category of food items available, followed by beverages either in pre-packaged or pre-made drinks and pre-packaged foods.

Table 3 presents the distribution of food items available in both school canteens based on 10 major food groups. As expected, it was a similar pattern of food groups found between primary and secondary school canteens, whereby grains and cereal-based products was the main food category available, followed by beverages, confectionary and sweet foods, fried snacks and fast foods, and animal-based food products. When these specific main food groups were re-classified as main staple food products, animal-based protein products, high fats and sweet foods, fruits and vegetables, and legume-based foods, it was found that high fats and sweet food products (23.4% and 24.1%) and sugary beverages (24.9% and 21.3%) were among the most common food products available besides the main staple products sold, in both primary or secondary school canteens. In contrast, fruit and vegetables (3.2% and 1.0 %), and legume-based products (0.8% and 1.9%) were the least common food items available. Comparisons of food-type distributions between primary and secondary school canteens showed quite a consistent pattern and trend for both school canteens, except for animal-based foods. A greater percentage of food items in this category was found in 12.1% of secondary schools vs. 6.7% of primary schools. Interestingly, there were no fruit items available in secondary school canteens, unlike in primary school canteens (2.4%).

The healthiness of foods was determined by the total sugar content of all pre-packaged foods and beverages based on the traffic-light nutrition rating system (Table 4). Out of 147 pre-packaged food items available in both school canteens, a total of 110 foods and beverages (74.8%) contained front-of-pack nutrient information on total sugar content. Overall, almost one-third of these pre-packaged food and beverage products (29.1%) received the red traffic-light rating as less healthy foods and beverages. As expected, confectionary and sweet foods (19.1%) and flavoured yogurt drink and fruit cordial drinks (10.0%) both exceeded the recommended sugar levels of >22.5 g per 100 g and >11.25 mL per 100 mL, respectively. In contrast, only one of these packaged foods (0.9%) was classified as a healthy food choice based on the total sugar content. When these pre-packaged food items were further classified based on the NOVA food processing classification criteria [28], all were classified as ultra-processed food and drink products (data not shown).

Table 5 shows the distribution of the prohibited food items and the recommended healthy food types. In general, there was a significant amount of prohibited foods and beverages sold at both primary and secondary school canteens. About a quarter of foods and beverages (26.8%) were classified as prohibited food items based on the new revised prohibited food list based on the Healthy School Canteen Management Guide [25]. Processed foods. such as burgers, sausages, nuggets, fish balls, and related foods, were considered the most popular food items in both canteens, followed by cream rolls, bread, and creamy biscuits, and pre-mixed cordial and syrup beverages. On the contrary, when these foods in both school canteens were further compared with the proposed recommended healthy food items and/or menus based on the Healthy Cafeteria Initiative [29], only 10.2% were classified as highly recommended healthy food choices in these canteens. Comparisons of food quality distribution showed that secondary school canteens offered higher amounts of prohibited foods compared to primary schools (28.3% vs. 24.9%). A similar pattern was found for the recommended healthy food items and choices in these canteens, whereby secondary school canteens offered slightly more healthy food items than primary school canteens (11.8% vs. 8.3%). Interestingly, most prohibited food items sold in these school canteens were considered ultra-processed food products based on the NOVA food processing classification. In contrast, most recommended healthy food items were classified as unprocessed or minimally processed foods.

## 4. Discussion

Schools are an important food environment to cultivate and promote healthy food choices and eating practices among children. Increasing the availability and attractiveness of a wide range of healthy food choices and, at the same time, restricting the availability of less healthy food that are relatively low in nutrients and high in sugars and fats is an important strategy [20,21]. Previous studies have indicated that food availability in school is one of the strongest determinants of food-eating choices among school-aged children and adolescents [24,30,31]. To the best of our knowledge, this study is the first to assess in detail, the distribution of foods and beverages in terms of types and quality assessed by several assessment criteria in both primary and secondary schools. The main findings of the present study indicate that despite main staple food products, high-fat and sweet food products, and sugary beverages being the most common foods and beverages stocked in these primary and secondary school canteens. In contrast, healthy food types, such as fruits and vegetables and legume-based products, were less common foods. This pattern is consistent with previous work conducted in primary school canteens [32]. Hence, the present findings reiterate the fact that the presence of more low nutrient, energy-dense foods, such as high fat and sweet foods and sugar-sweetened beverages, and fewer fruits, vegetables, and legume-based foods is still a major concern in these school canteens. This observation may be significantly associated with poor dietary eating choices [20,23,24,30]. For instance, students who had greater access to à la carte food products tend to consume more low-nutrient, energy-dense foods, such as sugar-sweetened beverages, and fewer fruits and vegetables [30,31]. If that would be the case, this could significantly influence the energy intake associated with high-calorie foods that are high in fats and sugars and consequently may lead to poor energy balance and the risk of excessive weight gain.

Most pre-packaged foods and beverages available in school canteens had considerably moderate to high content of sugars, with almost one-third of these pre-packaged food products classified as less healthy. Most were confectionary and sweet foods, flavoured yogurt drinks, and fruit cordial drinks with added sugars. Furthermore, about a quarter of the food items available in school canteens were classified as prohibited based on the new revised list of prohibited food and beverage items. This pattern of high-sugar content is consistent with previous studies in primary school canteens in Malaysia [32] and school meals reported in the United States [30,33]. The present findings emphasise that the dietary pattern of high sugar contents found in foods and beverages in school canteens is a major concern. The excessive consumption of sugars in children and adolescents is an emerging public health concern [34,35,36]. High intake of sugar in children and adolescents have been significantly associated with increased risk of dental caries [37], poor diet quality [38], excessive weight gain and obesity [34,36], and cardiometabolic disorders [39].

The consumption of ultra-processed foods has become more common worldwide, including in Malaysia [40]. A growing body of evidence has suggested that food processing levels could be used to display “very healthy” perspectives on the studied foods, whereby the pattern of ultra-processed food consumption might be a marker of a constellation of poor diet quality, in which the higher consumption of ultra-processed foods has been significantly associated with the greater intake of calories, sugars, fats, and sodium [41,42] and the increased risk of poor health outcomes in children and adolescents [43,44]. Analyses of the food quality of items available in both primary and secondary school canteens were further classified according to the NOVA food processing classification based on the extent and purpose of industrial food processing [28]. It is interestingly to note that most prohibited food items and high-sugar content pre-packaged food and beverages available in the present study were considered ultra-processed food products based on the NOVA food processing classification, whereas most recommended healthy food items were classified as unprocessed or minimally processed. Overall, the present results suggest that ultra-processed foods available in school canteens are associated with a higher proportion of unhealthy food consumption, in both primary and secondary school children, as found in previous studies in other countries [41,42]. This suggests that most ultra-processed food products are often high in sugar and fat content and are another important nutrition concern that needs greater attention, because unhealthy choices have become the most common food choice among school-aged children in school canteens.

Comparisons of food types and quality between primary and secondary school canteens found that the latter offered higher amounts of prohibited foods, as reflected by the fact that a higher proportion of high-fat foods such as the processed meat foods, cream rolls, bread, and creamy biscuits in the prohibited food list in secondary school canteens, which perhaps could possibly be attributed to high demand and food preference among secondary school children. Several plausible explanations could help to explain the presence of more food products that are high in calories, sugars, fats, and sodium foods in secondary school canteens. As children get older, they tend to spend large amount of time in school, and they are most likely to make more food purchases in school canteens because they are not allowed to leave the school grounds during school hours, as indicated by the total number of foods available in secondary school canteens. Secondly. they tend to have more independence and autonomy for more food choice behaviours because they might have had more disposable income (pocket money) at this age to purchase more foods compared to their younger peers. Lastly, they are also more susceptible to many influences that are beyond the family environment, such as from schools, the local community, mass media, and social media, as well as peer influence [16].

The present findings of the food availability pattern found more often in secondary school canteens than in primary school canteens are in line with a study comparing the food items offered between intermediate schools and elementary schools in the United States [31]. Moreover, the high availability of energy-dense foods tends to include foods that are also high in sugars, fats, and/or salt reported in these secondary school canteens, which could result in more frequent purchases of these foods. This could possibly be explained by the fact that adolescents tend to consume foods high in fats, sugars, and carbohydrates and low in fruits and vegetables, compared to their younger counterparts in schools [45,46]. Hence, food environment indeed influences student food choices and consumption [20,21]. These findings, together with the current study have revealed that adolescents tend to choose and consume less healthy food and an unbalanced diet when they are given more free choice. On the other hand, there were not many differences among the recommended healthy food items and choices or the healthiness of total sugar of pre-packaged foods across both primary and secondary school canteens. These findings have implications for schools and suggest actions that schools could use as “alternative” avenue to encourage healthier eating practices by providing a wide variety of healthy food choices when children get older and they are given more choices, because a healthy diet during school-age has significant implications for general health and nutritional well-being of their current life and later in life [2,3,7].

### Strengths and Limitations of the Study

The strengths of the present study were that all schools were recruited using random sampling based on the two main public-school categories available in order to get a more representative sample of school-aged children in terms of age and ethnicity in Kelantan, Malaysia. Secondly, the present study was carried out using direct observation of school canteens, in which detailed information of entire foods and beverages were collected. Additionally, the data collected were objective and not subject to researcher bias. In addition, the present results provide novel information on the presence of total sugars in the school food environment, in which detailed information on the helpfulness of pre-packaged foods and beverages in school canteens, and restrictions on the access to unhealthy food items and healthy school meals at school were also assessed. Findings from this present study should be useful to the government education and health agencies to develop and formulate more specific nutrition actions such as reducing sugar consumption and also for the proper planning of new healthy nutrition standards.

Nonetheless, this study also has some limitations. First, differences between students in terms of socio-demographic background could possibly influence their purchasing, consumption, and compensatory eating behaviours. Hence, further research is needed to determine the relationships between student food-eating practices, socio-demographic factors, and interactions with food environments in schools on the risks of excessive weight gain and cardio-metabolic disorder. Secondly, due to its cross-sectional observational design, the causality of associations cannot be established. Lastly, the present work only focused on the detailed distribution in terms of types and quality of foods and beverages served in school canteens as measurement indicators of the availability and accessibility of foods and beverages in the school food environment. It has been used as “measures” of food availability and accessibility in present schools because most students purchased foods solely in their school canteen because there were no vending machines allowed in the schools and, also, students were not allowed to go outside of the school during their time at school. It is generally known that there is no standardised methods of assessment developed up to the present time to assess the school food environment [22]. It is hoped that more comparable robust methods of assessment can be developed to determine and monitor the school food environment from across different sociodemographic and geographical regions across different countries, in order to better understanding the role of the school food environment on the development of obesity and disease-related risks among school-aged children and adolescents in the near future.

## 5. Conclusions

These findings indicate that, despite the Guidelines, a large number of unhealthy food items are being sold in school canteens. Hence, interventions, such as sustainable healthy school canteen menus, should be implemented to promote healthy food choices amongst school-aged children. Awareness of and interventions regarding healthy eating practices among students should be implemented in schools, such as effective nutrition intervention strategies with active partnerships with all relevant stakeholders, namely, school authorities, food operators, teachers, parents, and students, to develop and strengthen the implementation of healthy school nutrition promotion-related practices and policies. These include increasing the availability of healthy food options, such as fruits and vegetables, and/or restricting the availability of low-nutrient, energy-dense food products available, such as foods and beverages high in sugars and fats, in school settings, which may positively impact students’ dietary habits and their general nutritional and health well-being.

## Figures and Tables

**Table 1:** Classification of the total sugar content based on the UK Nutritional Traffic Light System.

	Recommended Total Sugar Level per 100 g of Food or 100 mL of Beverage		
	Green (Low)	Amber (Medium)	Red (High)
Food	≤5.00 g	>5.0 g to ≤22.50 g	>22.50 g or >27.0 g/portion
Beverage	≤2.50 g	>2.5 g to ≤11.25 g	>11.25 g or >13.5 g/portion

Food Standards Agency, UK (2007).

**Table 2:** General characteristics of food and beverages available in primary and secondary school canteens.

	Primary Schools (*n* = 253)	Secondary Schools (*n* = 315)
Foods, n	178	229
Beverages, n	75	86
Types of Food and Beverages	% (*n*)	
Cooked food ^a^	59.7 (151)	51.7 (163)
Pre-packaged food ^b^	7.5 (19)	15.6 (49)
Pre-ordered food ^c^	3.2 (8)	5.4 (17)
Pre-packaged beverages ^d^	15.0 (38)	13.0 (41)
Beverages (made in schools) ^e^	14.6 (37)	14.3 (45)

^a^ Food were heated before serving or sold in ready-to-eat form and these foods were usually baked, roasted, fried, broiled, or sautéed in advance.; ^b^ Foods that are sealed in a packaged form such as in box, bag, can or other container.; ^c^ Food that need to be placed an order before it is available for purchase.; ^d^ Beverages that are sealed in a box, bag, can or other container and usually sold in the grocery store in their packaged form.; ^e^ Beverages that are prepared before serving.

**Table 3:** Distribution of food and beverage types in both primary and secondary school canteens based on 10 food categories.

Food Categories	Primary Schools	Secondary Schools
	% (*n*)	
Grains and cereal products	35.6 (90)	32.7 (103)
Noodles/pasta based cooked dishes	47.8	49.5
Rice-based cooked dishes	31.1	28.2
Bread type products	21.1	10.7
Others	0	11.7
Beverages	24.9 (63)	21.3 (67)
Pre-made drink	58.7	67.2
Pre-packaged drink	41.3	32.8
Confectionary and sweet foods	11.9 (30)	13.3 (42)
Pre-packaged bakery wares or cookies	43.3	40.5
Sweet pastries	26.7	9.5
Traditional local deserts (*kuih*)	13.3	11.9
Pre-packed snacks	6.7	9.5
Pre-packed pudding dessert	6.7	4.8
Pre-packaged flavoured cream rolls	3.3	16.7
Pre-packed hard candy	0	7.1
Snack and fast foods	11.5 (29)	10.8 (34)
Potatoes fries	20.7	11.8
Fried chicken strips/popcorn chicken	17.2	14.7
Nuggets	13.8	14.7
Burgers	10.3	17.7
Sausages	3.5	17.7
Fried fish crackers (*keropok*)	13.8	11.8
Fried savoury filling fritters	13.8	2.9
Fried fish ball	3.5	5.9
Fried crab flavoured filament stick	3.5	2.9
Animal-based foods	6.7 (17)	12.1 (38)
Fish-based dish	52.9	44.7
Chicken-based dish	47.1	55.3
Milk and dairy products	4.7 (12)	6.0 (19)
Pre-packed flavoured yogurt drink	83.3	52.6
Pre-packed flavoured milk drink	16.7	42.1
Pre-packaged plain milk	0	5.3
Fruits	2.4 (6)	0
Fresh fruits (cut)	100.0	
Vegetables	0.8 (2)	1.0 (3)
Vegetables dishes	100.0	100.0
Legumes	0.8 (2)	1.9 (6)
Pre-packaged nuts and seeds	50.0	83.3
Soy-based products	50.0	16.7
Eggs	0.8 (2)	1.0 (3)
Egg-based dishes	100.0	100.0

**Table 4:** Distribution of total sugar contents based on pre-packaged food and beverage items in primary and secondary school canteens using the Traffic-Light Nutrition Rating System.

	Primary Schools			Secondary Schools			Combined Schools		
	Green ^a^ (*n* = 0)	Amber ^b^ (*n* = 36)	Red ^c^ (*n* = 16)	Green ^a^ (*n* = 1)	Amber ^b^ (*n* = 41)	Red ^c^ (*n* = 16)	Green ^a^ (*n* = 1)	Amber ^b^ (*n* = 77)	Red ^c^ (*n* = 32)
**Pre-packaged food**	0	7.7 (4)	19.2 (10)	1.7 (1)	10.3 (6)	19.0 (11)	0.9 (1)	9.1 (10)	19.1 (21)
Grain and cereal products	0	0	0	0	1.7 (1)	0	0	0.9 (1)	0
Nuts and legumes	0	1.9 (1)	0	0	1.7 (1)	0	0	1.8 (2)	0
Confectionary and sweet foods	0	5.8 (3)	19.2 (10)	1.7 (1)	6.9 (4)	19.0 (11)	0.9 (1)	6.4 (7)	19.1 (21)
**Pre-packaged beverages**	0	61.5 (32)	11.5 (6)	0	60.3 (35)	8.6 (5)	0	60.9 (67)	10.0 (11)
Flavoured milk	0	3.8 (2)	0	0	13.8 (8)	0	0	9.1 (10)	0
Flavoured yogurt drink	0	9.6 (5)	9.6 (5)	0	12.1 (7)	5.2 (3)	0	10.9 (12)	7.3 (8)
Fruit cordial drink	0	25.0 (13)	1.9 (1)	0	10.3 (6)	3.4 (2)	0	17.3 (19)	2.7 (3)
Herbal infusion beverages	0	9.6 (5)	0	0	3.4 (2)	0	0	6.4 (7)	0
Malted drink	0	0	0	0	3.4 (2)	0	0	1.8 (2)	0
Soybean-based beverages	0	5.8 (3)	0	0	3.4 (2)	0	0	4.5 (5)	0
Tea-based beverages	0	3.8 (2)	0	0	8.6 (5)	0	0	6.4 (7)	0
Carbonated flavored drinks	0	3.8 (2)	0	0	5.2 (3)	0	0	4.5 (5)	0

^a^ Low in total sugar contents and is a healthier choice; ^b^ Neither high nor low in total sugar contents; ^c^ High in sugar contents and is a less healthy food.

**Table 5:** Classification of foods and beverages available in school canteens based on the Healthy Cafeteria Guideline and the NOVA food processing classification system.

Food Items	Processed Food Group ^c^	Primary Schools	Secondary Schools	Combined Schools
		**% (*n*)**		
**Prohibited Food ^a^**		24.9 (63)	28.3 (89)	26.8 (152)
Burgers, sausages, nuggets, fish balls, flakes	G^4^	8.3 (21)	11.1 (35)	9.9 (56)
Cream rolls, breads, creamy biscuits	G^4^	4.0 (10)	6.0 (19)	5.1 (29)
Junk foods	G^4^	2.4 (6)	1.9 (6)	2.1 (12)
Cakes and donuts	G^4^	2.8 (7)	0.6 (2)	1.6 (9)
French fries	G^4^	1.6 (4)	1.3 (4)	1.4 (8)
Instant noodles	G^4^	0.4 (1)	1.0 (3)	0.7 (4)
Candy	G^4^	0	1.0 (3)	0.5 (3)
Sweet chocolate snacks	G^4^	0.4 (1)	0.6 (2)	0.5 (3)
Pre-mixed cordial and syrup drinks	G^4^	4.3 (11)	3.8 (12)	4.0 (23)
Carbonated flavoured drinks	G^4^	0.8 (2)	1.0 (3)	0.9 (5)
**Highly Recommended ^b^**		8.3 (21)	11.8 (37)	10.2 (58)
Whole grains and whole grains	G^4^	0	0.3 (1)	0.2 (1)
Fresh fruits and vegetables	G^1^	2.8 (7)	0.6 (2)	1.6 (9)
Fruit and vegetable juices	G^1^	0	1.3 (4)	0.7 (4)
Seafood, poultry, and eggs	G^1^	4.3 (11)	8.9 (28)	6.9 (39)
Low fat or skimmed milk	G^1^	0	0	0
Soybeans milk	G^1^	0.8 (2)	0.6 (2)	0.7 (4)
Steamed foods such as steamed corn	G^1^	0.4 (1)	0	0.2 (1)
Legume-based foods (tempeh, tofu)	G^1^	0	0	0

^a^ Classification based on the new revised list of prohibited food and beverage items in school canteens recommended in the Healthy School Canteen Management Guide (Ministry of Education Malaysia, 2017); ^b^ proposed food types based on the recommended food menu choices by the Healthy Cafeteria Initiative Program (Ministry of Health Malaysia, 2011); G^1^ = unprocessed and minimally processed foods and G^4^ = ultra-processed food.

## Data Availability

Request to access the datasets should be directed to lhfoo2012@gmail.com.
